# Kaposi Sarcoma: Almost Forgotten but Occasionally There

**DOI:** 10.5334/jbsr.3874

**Published:** 2025-03-18

**Authors:** Frederic Claerhoudt, Koen Mermuys, Jesse Marrannes

**Affiliations:** 1 Sint‑Jan General Hospital, Bruges, Belgium and Ghent University Hospital, Ghent, Belgium; 2 Sint‑Jan General Hospital, Bruges, Belgium

**Keywords:** kaposi sarcoma, HIV, oncology

## Abstract

*Teaching point:* Kaposi sarcoma is a rare disease most commonly occurring in patients with human immunodeficiency virus (HIV), in patients receiving immunosuppressants or in African patients. Radiological imaging has a role in facilitating the diagnosis and follow‑up, currently primarily with positron emission tomography–computed tomography (PET‑CT) (1).

## Case

A 41‑year‑old man was referred to the emergency department with high fever. There was a red and swollen left groin area. There were purple skin lesions on his legs and abdominal flanks.

A thoracic and abdominal computed tomography (CT) scan revealed multiple enlarged lymph nodes in the retroperitoneum and the left inguinal and iliac areas.

The on‑call dermatologist described the skin lesions as suggestive of Kaposi sarcoma (KS) and ran a human immunodeficiency virus (HIV) test and human herpesvirus‑8 (HHV8) test, which were both positive. A biopsy later confirmed the diagnosis of KS.

**Figure 1:**
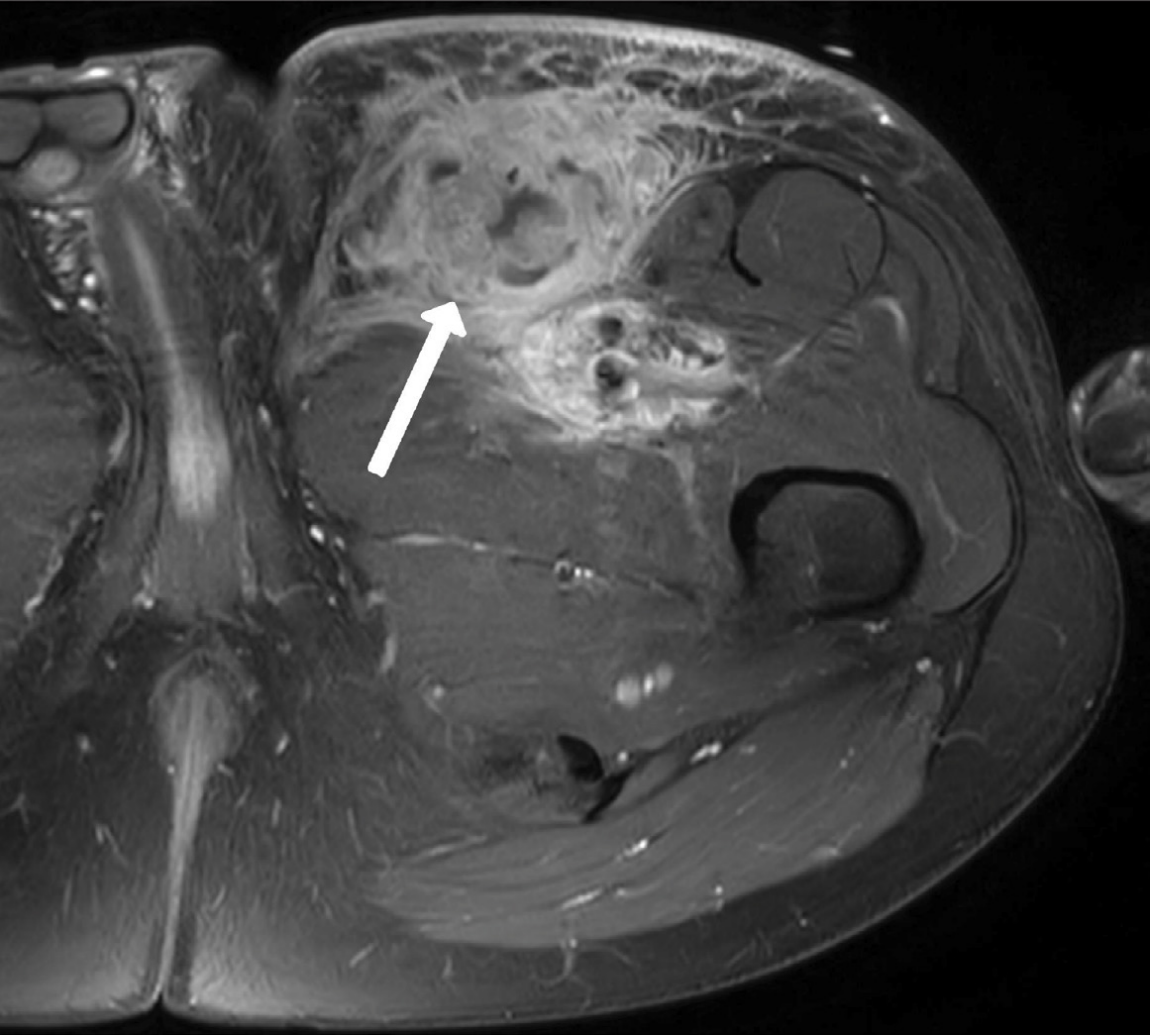
Enhancing mass in the left inguinal area with cystic/necrotic components.

**Figure 2:**
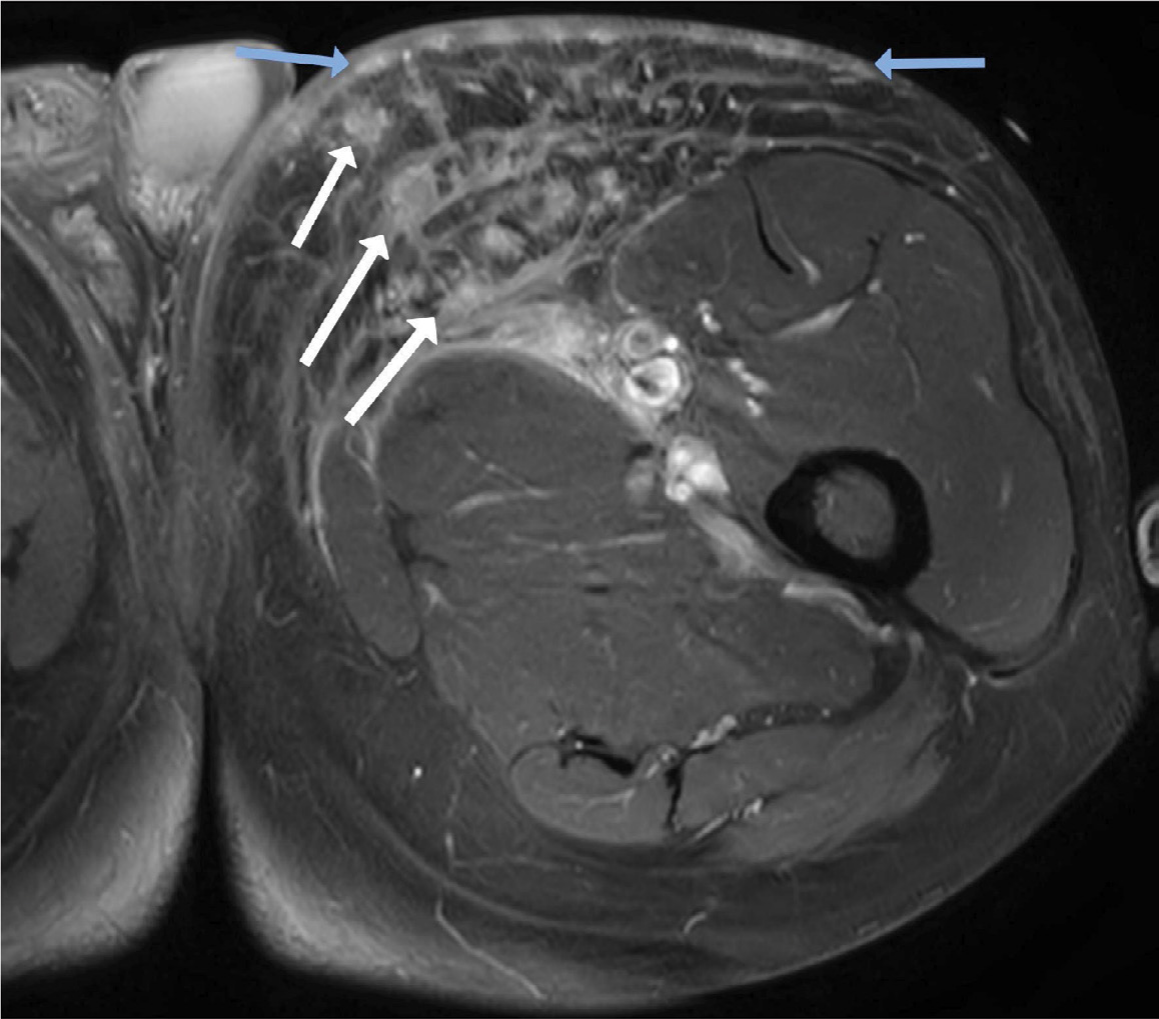
Multiple enhancing satellite lesions surrounding the mass (white arrows) and enhanced nodular skin thickening (blue arrows).

An MRI of the left upper leg demonstrated a voluminous heterogenous mass in the left inguinal area, surrounding the femoral vessels. The mass was iso‑intense to slightly hyperintense on T1‑weighted sequences and showed enhancement after intravenous contrast administration with cystic/necrotic components (Figure 1, arrow). There were also multiple satellite lesions in the left iliac area and in the subcutaneous fat of the left upper leg (Figure 2, white arrows). Finally, there was enhanced nodular skin thickening at the anterior aspect of left upper leg (Figure 2, blue arrows).

**Figure 3:**
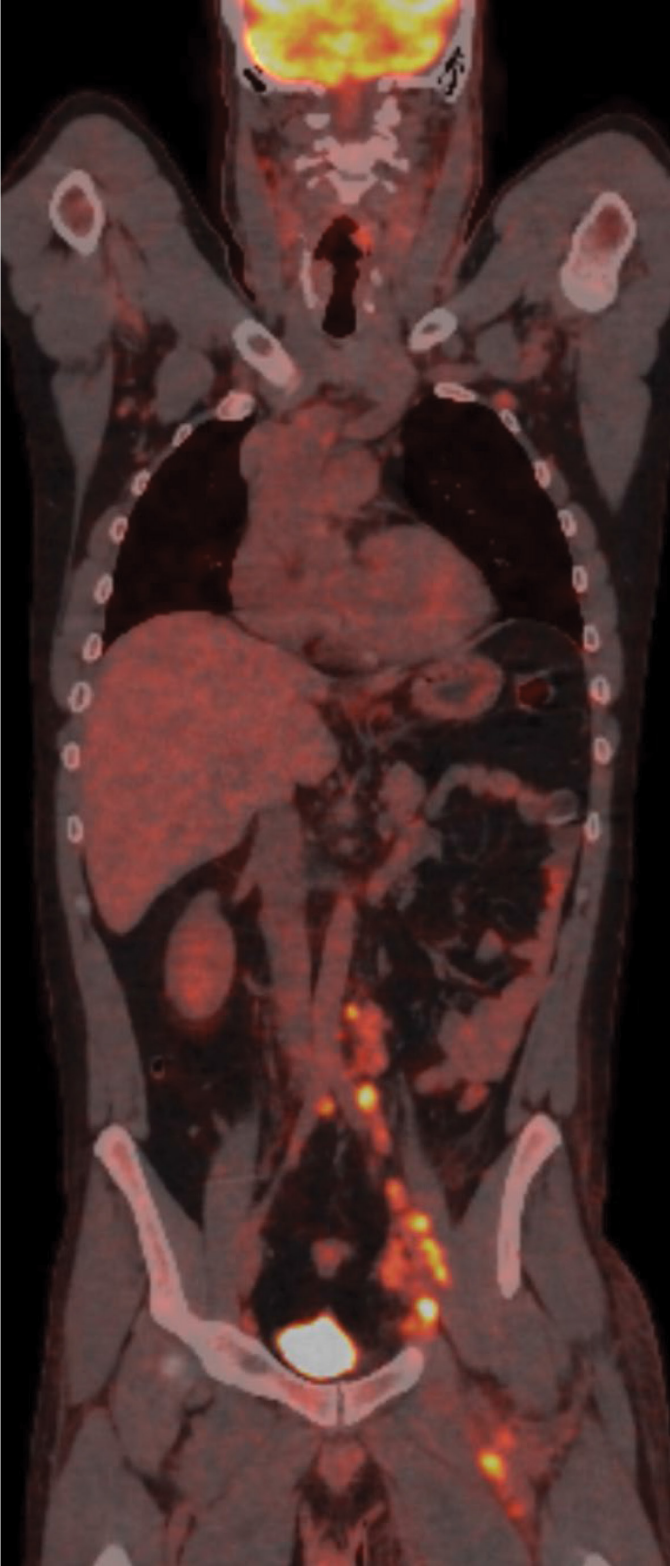
FDG‑PET‑CT scan showing multiple lymph nodes in the retroperitoneum, the left iliac and inguinal areas.

A fludeoxyglucose‑18–positron emission tomography–CT (FDG‑PET‑CT) scan revealed multiple lymph nodes in the retroperitoneum, in the left inguinal area and adjacent to the left iliac and femoral vessels (Figure 3).

## Comment

Kaposi sarcoma (KS) is a well‑known disease entity caused by HHV8 that can be further divided into four variants: classic KS (usually indolent), endemic KS most often found in Africa, iatrogenic KS and epidemic/HIV‑related KS. This case depicts the epidemic variant of KS. Epidemic KS usually occurs on the skin, viscera and/or lymph nodes and can be multifocal. The skin lesions can occur anywhere but have a propensity for involvement of the lower extremities, as in this case. Skin lesions tend to be hyperintense on T2‑weighted sequences and enhance after gadolinium administration. When only the skin and lymph nodes are involved, as in this case, the prognosis is usually favourable. Visceral KS may affect the lungs, GI system, liver, spleen and urogenital tract, warranting special attention at imaging. FDG‑PET‑CT is now commonly used for the staging of KS.

The incidence of KS is steadily decreasing owing to the high efficacy of active antiretroviral therapy (HAART) for acquired immune deficiency syndrome (AIDS) (1).

The differential diagnosis includes lymphoma, tuberculosis and angiosarcoma. Usually, lymphoma and tuberculosis present with little to no skin involvement.
